# Supplementary material for: Learning curve and surgical outcome of robotic assisted colorectal surgery with ERAS program
Source: Sci Rep. 2022 Nov 29;12:20566. doi: 10.1038/s41598-022-24665-w (PMC9709162; doi:10.1038/s41598-022-24665-w)
Supplement: Supplementary file 1 — Supplementary Information 1. [file 41598_2022_24665_MOESM1_ESM.zip › LSC Raw data-Ñ[▒K/081/P_ñw╝╨░O▒Kñσ.pdf]

[redacted]

Pathologic diagnosis:
1. Colon, terminal ileum, and appendix, right hemicolectomy --- a. Adenocarcinoma, moderately differentiated, arising from the background tubulovillous adenoma, of ascending colon. Tumor invaded to submucosa. The appendix, ileocecal valve, bilateral cut ends and mesocolic lymph nodes (0/23) are free of tumor. b. Status biopsy with granulation tissue formation and foreign body type giant cell reaction. c. Fibrous obliteration of appendix.
2. Liver, biopsy --- Hepatocellular carcinoma, moderately differentiated.

Ancillary study for diagnosis:
1. Recut and reticulin stain and mucicarmine (-) done for section A.
2. IHC stains for hepatocyte antigen (+ in tumor), glutamine synthase (diffuse + in tumor), and CK7 (- in tumor) done for section A.

Prognostic and predictive factor:
1. Tumor size: 3x1 cm.
2. Depth of invasion: Submucosa.
3. Histologic grade: G2: Moderately differentiated.
4. Angiolymphatic invasion: Present.
5. Perineural invasion: Not identified.
6. Tumor deposits (discontinuous extramural extension): Not identified.
7. Macroscopic tumor perforation: Not identified.
8. Surgical Margins: a) Proximal Margin: Uninvolved by invasive carcinoma, distance of tumor from margin: 13 cm. b) Distal Margin: Uninvolved by invasive carcinoma, distance of tumor from margin: 14 cm. c) Radial or Mesenteric Margin: Uninvolved by invasive carcinoma, distance of tumor from margin: 3 cm.
9. Lymph node status: Uninvolved (0/23).
10. Extranodal involvement: Not identified.
11. Tumor regression grading S/P CCRT: No known presurgical therapy.
12. Type of polyp in which invasive carcinoma arose: Tubulovillous adenoma.
13. Additional pathologic findings: Not identified.
14. Pathological TNM Stage: pT1N0(According to the eighth edition, American Joint Committee on Cancer Staging Guidelines for Tumors).
15. TNM descriptors: Not applicable.

Gross description:
The specimen consists of 1) a segment of colon, 20 cm in length, and 10 cm in proximal circumference, 5 cm in distal cut end circumference, and a segment of terminal ileum, 8 cm in length, and 4 cm in cut end circumference. An unremarkable appendix, 6 cm, is noted. A gray tan elevated and tumor with central tissue defect of biopsy, 3x1 cm, is noted,13 cm to the proximal cut end. On cross section, the tumor invaded to the submucosa with 3 cm to the radial margin grossly. there is also a clip site, 2 cm proximal from the main tumor, seen on the mucosa, no tumor seen grossly. The appendix, ileocecal valve, and both cut ends are free of tumor. 2) "liver", 6 pieces of tan soft tissue, up to 3x0.1x0.1 cm. Representative part for section: A) specimen 2 B) proximal cut end C) distal cut end D) radial margin E-F) clip site G-J) tumor K) ileocecal valve L) appendix M-P) lymph nodes.

Note:
1. This case has been peer reviewed by two doctors.
2. The diagnosis has been concurred in the intradepartment consensus meeting on 2020/07/01.
#T-59420_2 #M-81403_2 2 1105 B0000F
#T-C4560_2 #M-09183_2 0 1105 000000
#T-59200_2 #M-09183_2 0 1105 000000
#T-62000_2 #M-81703_2 2 1140 00CM0F

已電子簽章

敬告：本單僅供醫師參考使用，不具任何申請效力！！
請病患妥為保管，若不慎遺失需自負個人資料外洩責任！
